# Spin-Permutation Diabatization: A General Framework for Spin Localization and Exchange Coupling

**DOI:** 10.1021/acs.jctc.5c01904

**Published:** 2026-01-05

**Authors:** Alicia Omist, David Casanova

**Affiliations:** † 226245 Donostia International Physics Center (DIPC), 20018 Donostia, Euskadi, Spain; ‡ Polimero eta Material Aurreratuak: Fisika, Kimika eta Teknologia Saila, Kimika Fakultatea, Euskal Herriko Unibertsitatea (EHU), PK 1072, 20080 Donostia, Euskadi, Spain; § IKERBASQUE, Basque Foundation for Science, 48009 Bilbao, Euskadi, Spain

## Abstract

We present a spin-permutation diabatization strategy that transforms ab initio spin-pure eigenstates into spin-localized diabatic states, enabling direct mapping to spin-effective Hamiltonians without projection or orbital localization. The method provides both a real-space decomposition of electronic states in terms of localized spins and a straightforward evaluation of exchange couplings. Applications to several representative systems, including ethylene torsion, prototypical diradicals (benzynes, xylylenes, methylene), trimethylenebenzene triradical, singlet–triplet excited states of organic chromophores, and triplet-pair states in a tetracene dimer, demonstrate that the approach provides magnetic couplings and affords a clear physical interpretation of interacting spins. This general and conceptually transparent framework bridges ab initio electronic structure theory and spin models, and is expected to be especially valuable for systems with nontrivial distributions of unpaired electrons, such as delocalized or strongly correlated molecular magnets and spin-active chromophores.

## Graphical abstract

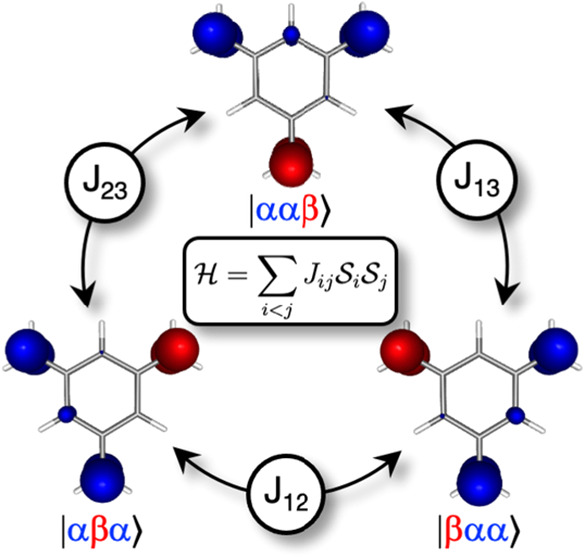

## 1 Introduction

Molecular systems hosting unpaired electrons have attracted sustained attention owing to their rich electronic structure, intriguing magnetic behavior, and wide-ranging potential applications, spanning from molecular spintronics^1,2^ and quantum information processing^3^ to organic magnetism^4^ and photocatalysis.^5^ Unlike most solid-state magnetic materials, magnetic molecules offer remarkable advantages such as synthetic tunability, chemical versatility, and the possibility of bottom-up design and scalability, enabling the rational tailoring of their magnetic and optical properties at the molecular level.

A central framework for describing the magnetic interactions between localized spins in molecules is provided by spin-effective Hamiltonians,^6^ which map the low-energy electronic structure of the molecule onto simplified spin models, such as the Heisenberg–Dirac-van Vleck (HDVV) Hamiltonian.^7–9^ In this context, exchange coupling constants quantify the magnetic interaction between spin centers and determine the relative energies of spin multiplets. Accurate computation of these couplings demands correlated ab initio approaches capable of capturing spin polarization and superexchange effects,^10^ including broken-symmetry DFT,^11^ multireference configuration interaction,^12^ complete active space methods,^13^ and spin-flip variants^14^ thereof. While these ab initio techniques can provide accurate values of exchange couplings, their results often lack straightforward interpretability in terms of localized electronic spins and their spatial interactions. This limitation motivates the development of qualitative frameworks and analysis tools to uncover the underlying physical mechanisms responsible for magnetic behavior. Such interpretative approaches might serve as an essential bridge between quantitative electronic-structure data and conceptual spin models, thereby facilitating the rational design of molecular architectures with tailored magnetic and photophysical properties.

In the realm of computational molecular photophysics, diabatization techniques based on the spatial localization of excitations offer a complementary perspective.^15–17^ By transforming adiabatic electronic states into localized and chemically intuitive diabatic excitations, diabatization enables the analysis of electronic transitions in complex systems (such as molecular dimers, oligomers, and aggregates) in terms of interacting diabatic configurations. This representation not only facilitates visualization of excited-state character and charge or exciton transfer processes but also provides a natural framework for computing electronic couplings that govern key photophysical mechanisms. However, conventional diabatization strategies typically rely on orbital-localization criteria, such as Boys localization,^18^ which maximizes the separation of charge centroids, or Edmiston-Ruedenberg localization,^19^ which maximizes the self-repulsion within each state, and therefore do not explicitly exploit the spin degrees of freedom that are fundamental to magnetic interactions.

In this work, we introduce a spin-permutation diabatization approach that constructs diabatic states based on the action of the spin-permutation operator. This new scheme enables a direct mapping of molecular wave functions onto a representation where individual spins are explicitly identified and coupled according to their mutual exchange interactions. We demonstrate that this formulation provides an intuitive and quantitative framework for analyzing magnetic and photophysical phenomena, ranging from simple diradicals and triradicals to excited-state spin couplings and multiexcitonic triplet-pair states.

## 2 Spin Permutation Localization Method

The spin permutation operator (*P̂*) appears as the nontrivial term in the Löwdin representation^20^ of the operator *Ŝ* ^2^

1 $${Ŝ}^{2}=\mathrm{P̂}+\frac{N\left(4-N\right)}{4}$$

where *N* is the total number of electrons and *P̂* is expressed as

2 $$\mathrm{P̂}=\sum_{i<j}{\mathrm{P̂}}_{\mathrm{ij}}^{\sigma }$$

where *P̂*_*ij*_^σ^ interchanges the spin coordinates of electrons *i* and *j*. In addition, the operator *P̂* can be used for the construction of spin eigenfunctions as linear combinations of elementary spin eigenfunctions of *Ŝ*_*z*_.^21^ Here our goal is just the opposite, i.e., the deconvolution of spin adapted eigenstates (eigenfunctions of *Ŝ* ^2^) in terms of electron spin states with local *S* = 1/2 spins. For that, we seek a unitary transformation **U** of *N*_*s*_ adiabatic states {Φ_*I*_} (eigenstates of *Ŝ* ^2^) into a diabatic representation {Ξ_*I*_}.

3 $$\Xi_{I}=\sum_{J}^{N_{s}}\Phi_{J}U_{\mathrm{JI}},I=1,...,N_{s}$$

Here, we define the rotation matrix **U** in eq 3 as the transformation that minimizes *self-spin exchange* by the minimization of the sum of squares of spin exchange expectation values

4 $$D\left(\Xi \right)=\sum_{I}^{N_{s}}\kappa_{\mathrm{II}}^{2}$$

where κ_*IJ*_ = ⟨Ξ_*I*_|*P̂*|Ξ_*J*_⟩.

Equation 4 resembles the sum of squares criterion employed in the Boys localization procedure,^18^ and it is also analogous to the Edmiston and Ruedenberg scheme^19^ for obtaining localized orbitals through the maximization of the self-repulsion energy. Notice that minimization of eq 4 is equivalent to maximization of the sum of interstate spin exchange terms, that is, off-diagonal terms κ_*IJ*_ with *I* ≠ *J*.^22^

Diabatic states are obtained by the successive transformation of pairs of states by applying a rotation of angle γ

5 $$\Xi_{1}=\Phi_{1}\cos\gamma +\Phi_{2}\sin\gamma$$

6 $$\Xi_{2}=-\Phi_{1}\sin\gamma +\Phi_{2}\cos\gamma$$

Substitution of eqs 5 and 6 into eq 4 yields^23^

7 $$D\left(\Xi \right)=D\left(\Phi \right)+A_{12}+{\left(A_{12}^{2}+B_{12}^{2}\right)}^{1/2}\cos\left[4\left(\gamma -\alpha \right)\right]$$

where

8 $$D\left(\Phi \right)=\sum_{I}^{N_{s}}\left\langle \Phi_{I}\right|\mathrm{P̂}\left|\Phi_{I}\right\rangle$$

9 $$A_{12}=\kappa_{12}^{2}-\frac{1}{4}{\left(\kappa_{11}-\kappa_{22}\right)}^{2}$$

10 $$B_{12}=\kappa_{12}\left(\kappa_{11}-\kappa_{22}\right)$$

11 $$\cos4\alpha =\frac{-A_{12}}{{\left(A_{12}^{2}+B_{12}^{2}\right)}^{1/2}}$$

Then, the minimum of eq 7 is obtained for γ values in the series

12 $$\gamma_{\min}=\frac{2n+1}{4}\pi +\alpha ,\;\mathrm{for}\;n=0,1,2...$$

The diabatization of *N*_*s*_ states is achieved by the successive applications of two-state transformations until a convergence criterion is satisfied.

### 2.1 Two-Spin Model

To illustrate the action of our spin diabatization strategy, we consider the simple case of a two-spin system, with spin singlet and triplet eigenstates

13 $$\left|S\right\rangle =\frac{1}{\sqrt{2}}\left\{\left|\alpha \beta \right\rangle -\left|\beta \alpha \right\rangle \right\}$$

14 $$\left|T\right\rangle =\frac{1}{\sqrt{2}}\left\{\left|\alpha \beta \right\rangle +\left|\beta \alpha \right\rangle \right\}$$

In this basis, κ_*IJ*_ = (−1)^*S*+1^δ_*IJ*_ and the transformation minimizing *D*(Ξ) is a rotation with γ_min_ = π/2, generating the two configurations with localized spins, that is, the two *M*_*z*_ = 0 elementary spin eigenfunctions of *Ŝ*_*z*_

15 $$\left|\Xi_{1}\right\rangle =\left|\alpha \beta \right\rangle$$

16 $$\left|\Xi_{2}\right\rangle =\left|\beta \alpha \right\rangle$$

In the diabatic basis the Hamiltonian terms are obtained as

17 $$E\left(\Xi_{1}\right)=E\left(\Xi_{2}\right)=\frac{E\left(T\right)+E\left(S\right)}{2}$$

18 $$\left\langle \Xi_{1}\right|H\left|\Xi_{2}\right\rangle =\frac{E\left(T\right)-E\left(S\right)}{2}$$

with eqs 17 and 18 corresponding to the diagonal and off-diagonal terms, respectively.

### 2.2 Mapping Diabatic and Spin Hamiltonians

The diabatization strategy described above provides a natural framework for defining the eigenstates of molecular systems in terms of interacting spins. This approach enables a direct correspondence between ab initio molecular Hamiltonians and effective spin Hamiltonians, such as the HDVV model (eq 19), which describes the magnetic interactions between localized spin centers. Furthermore, the characterization of diabatic states inherently yields a spatial description of spin localization and magnetic coupling, without requiring predefined localization schemes or projection techniques.^24,25^ This feature makes it possible to work with spin states that possess well-defined spatial localization, as naturally obtained through spin-permutation-based diabatization of electronic eigenstates. Such a spatially resolved picture provides a clearer physical interpretation of the resulting spin states and is particularly valuable in systems where spin localization is nontrivial, such as in π-conjugated magnetic molecules.

19 $${Ĥ}_{\mathrm{HDVV}}=\sum_{i<j}J_{\mathrm{ij}}{Ŝ}_{i}\cdot {Ŝ}_{j}$$

The diabatization scheme based on the spin-permutation operator offers a systematic and general procedure to generate a basis of localized spin states, in which the spin-α and spin-β unpaired electrons are distributed on specific molecular regions. This framework can be applied to any number of spin centers and arbitrary interaction topologies. By comparing the matrix elements of the diabatic Hamiltonian with those of the effective HDVV model, the spin-exchange coupling constants (*J*_*ij*_) can be readily extracted. When the number of independent elements in the diabatic Hamiltonian matches the number of exchange parameters, the *J*_*ij*_ values follow directly from analytical comparison. In cases where the diabatic manifold contains additional degrees of freedom, the exchange couplings can instead be obtained through a fitting procedure. For example, in the case of spin-1/2 functions, the low-spin blocks of the effective HDVV Hamiltonian for two- and three-spin systems in this diabatic basis are given by

20 $$H_{\mathrm{HDVV}}^{\left(2,0\right)}=\frac{J}{2}\left[\begin{array}{ll} -1/2 & 1\\ 1 & -1/2 \end{array}\right]$$

21 $$H_{\mathrm{HDVV}}^{\left(3,1/2\right)}=\frac{1}{2}\left[\begin{array}{lll} \left(J_{12}-J_{23}-J_{13}\right)/2 & J_{23} & J_{13}\\ J_{23} & \left(-J_{12}-J_{23}+J_{13}\right)/2 & J_{12}\\ J_{13} & J_{12} & \left(-J_{12}+J_{23}-J_{13}\right)/2 \end{array}\right]$$

where the superscript (*m, n*) indicates a system of *m* coupled spins with a total spin projection *S*_*z*_ = *n*. The low-spin blocks of the two Hamiltonians are expressed in the |*αβ*⟩, |*βα*⟩ and |*ααβ*⟩, |*αβα*⟩, |*βαα*⟩ bases, respectively.

Finally, we note that the accuracy of the computed spin-exchange interactions depends on the quality of the underlying electronic structure method, while their physical interpretation follows directly from the associated diabatic eigenfunctions {Ξ_*I*_}.

It is worth mentioning that the spin-diabatization scheme could also be employed alongside more general exchange Hamiltonians, including anisotropic terms such as the Dzyaloshinskii-Moriya interaction. However, these require the explicit treatment of relativistic effects and lie beyond the scope of the present study. Their incorporation will be explored in future work.

## 3 Computational Details

Molecular geometries used in Section 4, together with details of the methods employed for their optimization, are provided in the Supporting Information. Electronic structure calculations of the eigenstates of the molecular Hamiltonian were performed using the restricted active space configuration interaction (RASCI) method.^14,26–28^ Unless otherwise specified, RASCI calculations were carried out without freezing any orbitals, thus treating all orbitals as active, and within the hole and particle approximation, that is, including all possible configurations with up to one hole in the RAS1 subspace or one particle in the RAS3 subspace. In all cases, we employed a minimal fully correlated RAS2 space, that is, two electrons in two orbitals for ethylene, molecular diradicals (benzynes, xylylenes, and methylene), and in the diabatization of excited states (Section 4.6), three electrons in three orbitals in the trimethylenebenzene triradical, and four electrons in four orbitals in the coupling of spin-triplet states (Section 4.7). In general, RASCI wave functions of the target states were derived using a high-spin restricted open-shell Hartree–Fock (ROHF) reference configuration in combination with a spin-flip excitation operator (RAS­(*h*,*p*)-SF), except in Section 4.6, in which the singlet RHF solution was used instead. For simplicity, all calculations employed the 6-31G basis set. Quantum-chemical computations were performed with a development version of the Q-Chem software package.^29^

The state diabatization strategy described in Section 2 was carried out using iterative Jacobi-type two-state rotations, and the procedure was terminated when either the maximum rotation magnitude within a sweep fell below 10^–8^ or the accumulated rotation matrix became symmetric to within a tolerance of 10^–14^, indicating numerical self-consistency of the diabatic transformation.

## 4 Results and Discussion

In this section, we present a series of illustrative examples showcasing the performance and versatility of the spin-permutation diabatization scheme in describing molecular systems in terms of two or three interacting electronic spins. We first examine the evolution of diabatic states along the torsional coordinate of ethylene, followed by the characterization of several prototypical diradicals (benzynes, xylylenes, and methylene) and a representative triradical, trimethylenebenzene. Subsequently, we analyze the relationship between the lowest singlet–triplet energy gaps and the nature of the underlying diabatic states in three organic chromophores. Finally, we investigate the diabatic states arising from coupled triplet–pair configurations in a noncovalent tetracene dimer. As a proof-of-concept demonstration of the method’s applicability beyond purely organic systems, we further include in the Supporting Information an illustrative characterization of the dinuclear [Cu_2_Cl_6_]^2–^ complex (Section S2.2).

### 4.1 Ethylene torsion

As a first illustration of the capabilities of our spin diabatization scheme, we examine the coupling between the two 2p*_z_* electrons in ethylene as a function of the torsion around the C=C bond (Figure 1). The diabatization is carried out between the ground-state singlet and the first triplet state. Similar analysis of the σ-bond dissociation in H_2_ can be found in the Supporting Information (Section S2.1).

**Figure 1:**
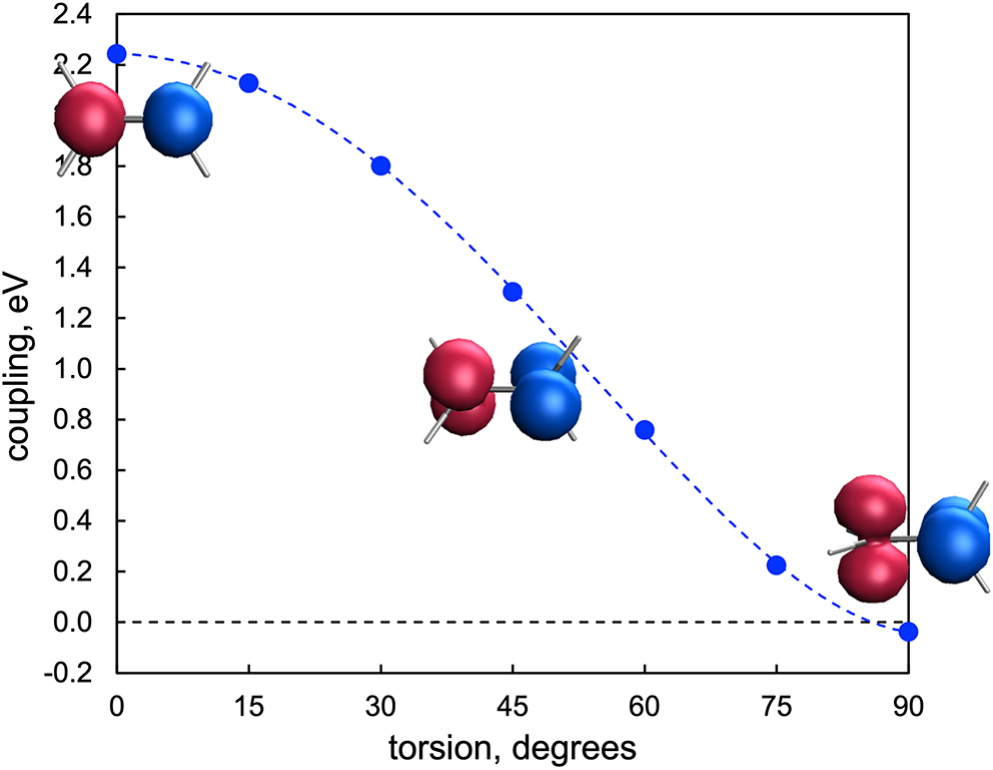
Electronic coupling (in eV) between the two diabatic states obtained from the *S*_0_ and *T*_1_ along the ethylenes molecular torsion. Inset: spin density of one of the diabatic states at 0°, 45°, and 90° torsion. The other diabat present the equivalent spin density distribution with interchanged α – β densities. Isovalue: 0.02 bohr^–3^.

At the equilibrium *D*_2*h*_ geometry, the *S*_0_ state corresponds to the double occupancy of the π-bonding orbital (the in-phase combination of the two 2p*_z_* atomic orbitals), while the *T*_1_ state involves single occupancies of the π and π* orbitals. The strong computed coupling at this planar structure reflects the robust π-interaction between the carbon atoms. As the molecule undergoes torsion around the C=C bond, the π-bond weakens, while the σ-framework remains largely intact, leading to a progressive reduction of both the singlet–triplet energy gap and the diabatic coupling. At a 90° torsion angle, the triplet state becomes lower in energy than the singlet, as indicated by the small and negative coupling value. Along the entire torsional coordinate, the diabatic states can be described as the |*αβ*⟩ and |*βα*⟩ broken-symmetry configurations, where the spin-α and spin-β electron densities are localized on opposite sp^2^ carbons and oriented consistently with the molecular torsion.

### 4.2 Benzynes: σ,σ-Diradicals

We apply the diabatization approach to a representative family of σ,σ-diradicals: the *ortho*-, *meta*-, and *para*-benzyne isomers. This molecular series provides an ideal framework to examine how spin localization and exchange interactions depend on the relative positions of the two unpaired electrons localized on the radical carbon atoms within the benzene ring. For each compound, the two diabatic states were derived from the lowest singlet and triplet eigenstates, namely the singlet ground state ^1^ *A*_1_ in *C*_2*v*_ symmetry (for *o*- and *m*-benzyne) and ^1^ *A*_*g*_ in *D*_2*h*_ symmetry (for *p*-benzyne), together with the corresponding lowest triplet excited states ^3^ *B*_2_ (*ortho* and *meta*) and ^3^ *B*_1*u*_ (*para*).

Spin-density plots of the diabatic states (Figure 2) illustrate how the unpaired electrons localize in the three benzyne isomers, reflecting the relative positions of the radical carbon atoms. The computed singlet–triplet energy gaps of 2.02, 1.47, and 0.11 eV for *o*-, *m*- and *p*-benzyne, respectively, are in good agreement with the highly accurate reference values reported by Vu et al.,^30^ especially considering the modest wave function expansion employed (see Section 3). These results can be rationalized in terms of through-space magnetic interactions between the spin centers located at the 1,2- (*ortho*), 1,3- (*meta*) and 1,4-positions (*para*). As the distance between the radical centers increases from *ortho* to *para*, the magnetic coupling weakens, resulting in a progressive decrease of the singlet–triplet energy gap.

**Figure 2:**
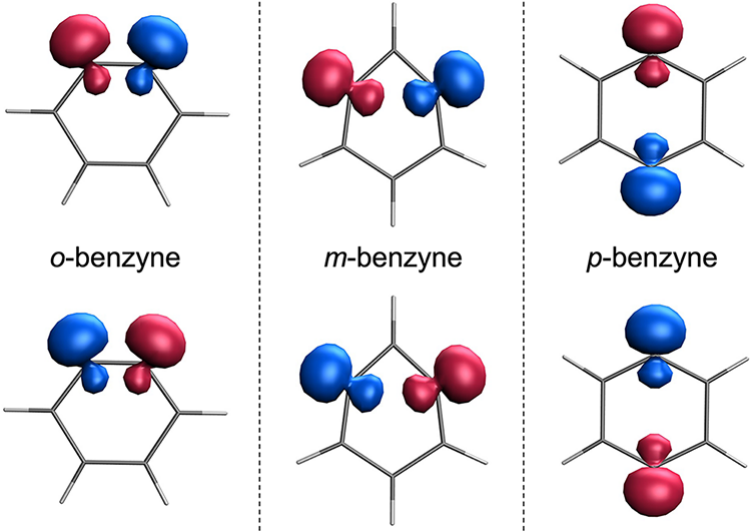
Spin density of the two diabatic states obtained for *o*-benzyne (left), *m*-benzyne (center), and *p*-benzyne (right) computed at the RAS­(*h*,*p*)-SF/6-31G level. Isovalue of 0.02 bohr^–3^.

### 4.3 Xylylenes: π,π-Diradicals

Similarly, we now show the capabilities of the spin-permutation diabatization method to generate spin states in the xylylenes (quinodimethanes) family, holding two unpaired π-electrons, one on each of the two methylene units (Figure 3).

**Figure 3:**
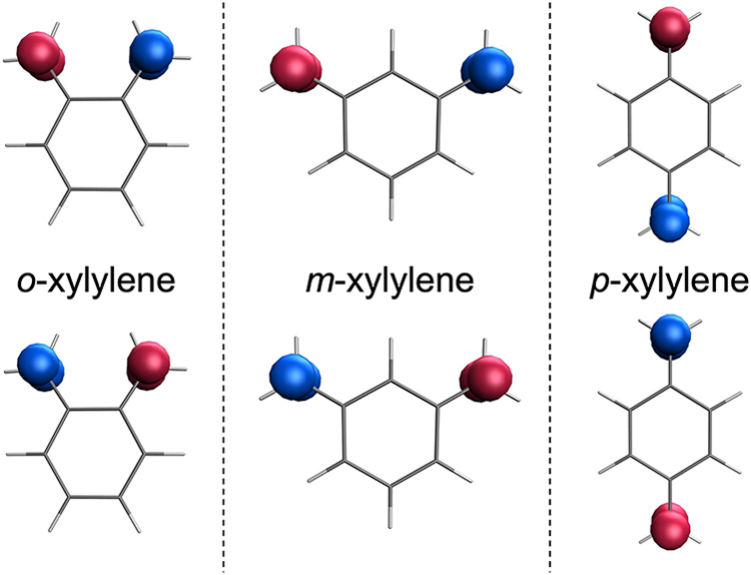
Spin density of the two diabatic states obtained for *o*-xylylene (left), *m*-xylylene (center), and *p*-xylylene (right) computed at the RAS­(*h*,*p*)-SF/6-31G level. Isovalue of 0.02 bohr^–3^.

*o*- and *p*-xylylene are Kekulé disjoint radicals with a singlet ground state essentially due to the spin-polarization mechanism,^31,32^ which can be described as a resonance between two closed-shell structures involving the nearly degenerate frontier π-orbitals. Their diradical character originates from quinoidal conjugation, which effectively restores aromaticity within the benzene ring.

In contrast, *m*-xylylene is a non-Kekulé diradical with a triplet ground state described as two electrons occupy two degenerate nonbonding molecular orbitals, as recovered by our calculations (Δ*E* = −0.20 eV) and in agreement with a variety of electronic structure calculations.^33–36^

Within the dimethylenebenzene series, the interaction between the unpaired electrons is mediated by π-conjugation (a through-bond mechanism), leading to spin-exchange coupling constants that decrease in the order *para* > *ortho* > *meta*. In the *para* isomer, the conjugated π-system of the benzene ring efficiently mediates the interaction between the two unpaired electrons, providing additional stabilization of the singlet state. Conversely, the *ortho* isomer is significantly destabilized due to both steric crowding and electronic repulsion between the closely spaced methylene substituents, which weakens the spin exchange. Finally, the *meta* isomer lacks an efficient pathway for through-ring conjugation, which not only reduces the magnitude of the spin-exchange interaction but also reverses its sign, leading to a ferromagnetic coupling and a triplet ground state.

### 4.4 State-Dependent Diabatizations in Methylene

The ground state of methylene (CH_2_) is a triplet (*X̃* ^3^ *B*_1_), in which the two unpaired electrons occupy the 3*a*_1_ and 1*b*_1_ molecular orbitals. These orbitals are primarily derived from the carbon 2p*_z_* orbital (oriented in-plane along the *C*_2_ symmetry axis) and the 2p_*x*_ orbital (oriented out of plane), respectively. As originally discussed by Salem and Rowland,^37^ CH_2_ features three low-lying singlet states of varying diradical character (*ã* ^1^ *A*_1_, *b̃* ^1^ *B*_1_, and *c̃* ^1^ *A*_1_), which arise from different electronic occupancies of these two frontier orbitals

22 $$\left|{ã}^{1}A_{1}\right\rangle \approx \lambda \left|3a_{1}\overset{®}{3a_{1}}\right\rangle -\sqrt{1-\lambda^{2}}\left|1b_{1}\overset{®}{1b_{1}}\right\rangle$$

23 $$\left|{\mathrm{b̃}}^{1}B_{1}\right\rangle \approx \frac{1}{\sqrt{2}}\left(\left|3a_{1}\overset{®}{1b_{1}}\right\rangle +\left|1b_{1}\overset{®}{3a_{1}}\right\rangle \right)$$

24 $$\left|{\mathrm{c̃}}^{1}A_{1}\right\rangle \approx \sqrt{1-\lambda^{2}}\left|3a_{1}\overset{®}{3a_{1}}\right\rangle +\lambda \left|1b_{1}\overset{®}{1b_{1}}\right\rangle$$

where the over bar indicates spin-β orbital, the parameter λ determines the character of the ^1^ *A*_1_ states, and its value strongly depends on the molecular geometry.

We apply the spin-permutation diabatization approach to the methylene diradical to analyze the three low-lying singlet states as resulting from the coupling of two unpaired electrons. To this end, we perform two-state diabatizations between the *X̃* ^3^ *B*_1_ ground state and each of the excited singlets, thereby enabling a description of each case in terms of the interaction between two diabatic states characterized by two unpaired electrons.

The *b̃* ^1^ *B*_1_ state is the second excited state of CH_2_, with a wave function that predominantly corresponds to the spin-singlet-adapted linear combination of single occupancies of the two frontier orbitals (eq 23). Consequently, as in the two-spin model discussed in Section 2.1, the diabatic states obtained from the spin-permutation diabatization of the triplet and singlet *B*_1_ states essentially correspond to $\left|3a_{1}\overset{®}{1b_{1}}\right\rangle$ and $\left|1b_{1}\overset{®}{3a_{1}}\right\rangle$ , that is, configurations with spin-up and spin-down electrons localized in the 3*a*_1_ and 1*b*_1_ orbitals, respectively, as illustrated by their spin-density plots (Figure 4). The computed coupling between these two diabatic states amounts to 1.00 eV, which is in agreement with the exchange integral between the 3*a*_1_ and 1*b*_1_ orbitals (*K* = 1.12 eV), and close to highly accurate estimates of the triplet-singlet vertical excitation energy.^38^

**Figure 4:**
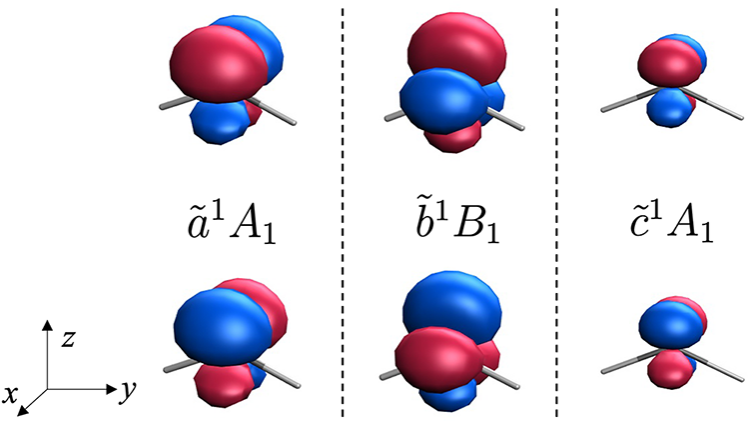
Spin density of the two diabatic states obtained from the diabatization of the triplet ground state of methylene and states *ã* ^1^ *A*_1_ (left), *b̃* ^1^ *B*_1_ (center), and *c̃* ^1^ *A*_1_ (right) computed at the RAS­(*h*,*p*)-SF/6-31G level. Isovalue of 0.02 bohr^–3^.

At the ground-state geometry, the *ã* ^1^ *A*_1_ state exhibits a pronounced closed-shell character, corresponding to a doubly occupied 3*a*_1_ orbital, as captured by our RAS-SF calculations (λ = 0.93). In contrast, the higher-lying *c̃* ^1^ *A*_1_ state is primarily characterized by the double occupation of the 1*b*_1_ orbital. Diabatizations of the ground-state triplet with the ^1^ *A*_1_ states yield diabatic states featuring single electron occupancies in orbitals formed as linear combinations of the two frontier orbitals, 3*a*_1_ ± 1*b*_1_. These correspond to orbitals oriented along the diagonals of the *xz*-plane, mainly composed of the 2p_*z*_ ± 2p_*x*_ combinations of carbon atomic orbitals (Figure 4). Although the diabatic spin densities display the same spatial pattern for both *ã* ^1^ *A*_1_ and *c̃* ^1^ *A*_1_, those of the *c̃* ^1^ *A*_1_ state appear attenuated relative to *ã* ^1^ *A*_1_. This attenuation reflects the reduced contribution of ionic configurations in the *ã* ^1^ *A*_1_ state, arising from the out-of-phase combination of the two closed-shell configurations (eq 22). Such a difference also accounts for the larger triplet-singlet energy gap of *c̃* ^1^ *A*_1_, and, equivalently, for its much stronger diabatic coupling (0.8 and 2.0 eV for *ã* ^1^ *A*_1_ and *c̃* ^1^ *A*_1_, respectively).

### 4.5 1,3,5-Trimethylenebenzene: πππ-Triradical

1,3,5-Trimethylenebenzene (TMB) is a paradigmatic example of a non-Kekulé triradical with a quartet (*S* = 3/2) ground state. In this molecule, the three unpaired π electrons are mainly localized on the methylene carbon atoms at the 1, 3, and 5 positions of the benzene ring. Because these unpaired electrons occupy nondisjoint π orbitals, their spin densities partially overlap, and the *meta* relative arrangement between each spin pair, analogous to that in *m*-xylylene, favors a ferromagnetic exchange interaction among the spin centers, ultimately stabilizing the high-spin quartet state.

We apply the spin diabatization approach to TMB at its optimized *D*_3*h*_ ground-state geometry. In this case, the diabatization is performed over the three lowest adiabatic states, namely, the quartet ground state (X^4^ *A*_2_^″^) and the doubly degenerate excited doublet state (1^2^ *E*″), to construct a three-state diabatic Hamiltonian. The resulting diabatic states correspond directly to the three neutral spin configurations, |*ααβ*⟩, |*αβα*⟩, and |*βαα*⟩, each representing a distinct alignment of the three localized spins on the methylene carbon radicals. The spin density plots of the three diabatic states (Figure 5) provide a clear visualization of the spin localization and coupling patterns among the three methylene carbons corresponding to the mentioned spin-configuration basis.

**Figure 5:**
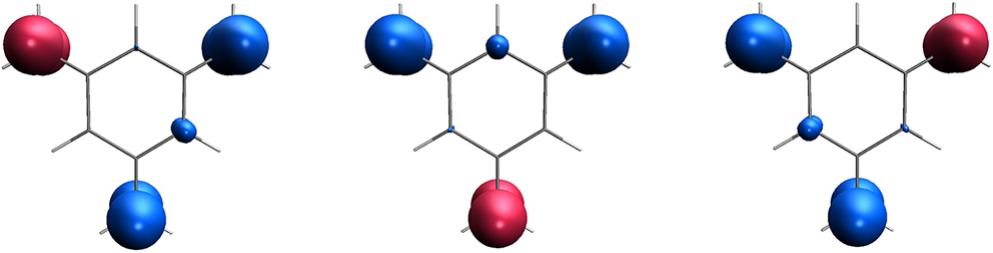
Spin density of the three diabatic states obtained from the diabatization of the X^4^ *A*_2_^″^ and 1^2^ *E*″ states of TMB computed at the RAS­(*h*,*p*)-SF/6-31G level. Isovalue of 0.01 bohr^–3^.

Analysis of the spin-exchange couplings, obtained by comparing the diabatic Hamiltonian with eq 21 under the assumption of 3-fold symmetry (*J*_12_ = *J*_13_ = *J*_23_), reveals a ferromagnetic interaction of approximately 4.6 kcal/mol. This value is in good agreement with previously reported highly correlated calculations,^39^ especially considering the modest basis set employed in the present work.

### 4.6 Excited State Diabatization

The relative energy between the lowest triplet (*T*_1_) and singlet (*S*_1_) excited states is a key factor governing the photophysical behavior and optoelectronic performance of molecular systems. Prominent examples include cases where *E*(*S*_1_) ≳ 2*E*(*T*_1_), *E*(*S*_1_) ≲ 2*E*(*T*_1_), and *E*(*S*_1_) ≈ *E*(*T*_1_), which are, respectively, relevant to singlet fission (SF),^40,41^ triplet–triplet annihilation (TTA),^42^ and thermally activated delayed fluorescence (TADF).^43^

In most organic chromophores, the *S*_1_ and *T*_1_ states predominantly arise from the same single-electron excitation, typically the HOMO-to-LUMO transition, with *T*_1_ lying lower in energy due to the exchange interaction between the involved orbitals. Electron correlation effects can, however, play a significant role in modulating this energy difference.^44^

The permutation diabatization scheme provides a useful framework to describe such excited states in terms of interacting diabatic components. This representation enables a spatial analysis of the underlying charge and spin distributions, offering insight into the nature and relative energies of *S*_1_ and *T*_1_. To illustrate the application of our scheme to excited states, we apply the diabatization method to the lowest singlet and triplet excited states of three representative organic chromophores, naphthalene (Nap), *p*-nitroaniline (PNA), and dicarbonyl klumpen triphenylamine (DiKTa), which exemplify distinct types of electronic excitation behavior. Naphthalene is the smallest polycyclic aromatic hydrocarbon (PAH) with *D*_2*h*_ symmetry. Its lowest-lying triplet and singlet excited states both belong to the *B*_2*u*_ irreducible representation and exhibit a ππ* character dominated by the HOMO→LUMO transition. The PNA chromophore adopts a fully planar geometry with *C*_2*v*_ symmetry. The difference in electronegativity between the electron-donating amino (NH_2_) group and electron-accepting nitro (NO_2_) gives rise to a pronounced charge-transfer (CT) character to the lowest-lying singlet (1^1^ *A*_1_) and triplet (1^3^A_1_) excited states, corresponding to a ππ* excitation from the HOMO, largely localized on the NH_2_ fragment, to the LUMO centered on the NO_2_ group. DiKTa is a multiresonant TADF (MR-TADF) emitter built on a rigid, fully fused aromatic scaffold that incorporates a single nitrogen donor atom together with two carbonyl acceptor functionalities. Because of the multiresonant framework, its lowest singlet and triplet excited states are characterized by so-called short-range CT (SRCT) transitions: the electron density alternates between adjacent atoms or moieties within the fused core, thereby enabling both a small singlet–triplet energy gap and narrow band emission.

The calculated excited-state spin–exchange coupling decreases in the order naphthalene > PNA > DiKta, mirroring the trend observed for the *T*_1_/*S*_1_ energy gaps. This behavior can be rationalized by examining the nature of the corresponding diabatic states. Diabatic states in which the spin-α and spin-β electrons exhibit similar spatial distributions show significant overlap between the two spin populations, leading to strong spin–exchange interactions and nearly vanishing net (diabatic) spin densities. Conversely, diabatic states with spatially separated spin distributions display weaker exchange couplings and smaller *T*_1_/*S*_1_ gaps. To quantify the degree of spin localization in the diabatic states, we evaluate the absolute spin population (*S*_abs_), defined as the sum of absolute atomic spin populations

25 $$S_{\mathrm{abs}}=\sum_{A}\left|P_{A}^{\alpha }-P_{A}^{\beta }\right|$$

The computed diabatic couplings and absolute spin populations for naphthalene, PNA, and DiKta are summarized in Table 1.

**Table 1:** Electronic Couplings (in eV) and Absolute Spin Populations of Naphthalene (Nap), PNA and DiKTa Molecules Computed at the RAS­(*h*,*p*)/6-31G Level with a RAS2 Space with Two Electrons in the Two Frontier Orbitals (HOMO and LUMO) in the RHF Singlet State

molecule	coupling	*S*_abs_
Nap	1.42	0.01
PNA	0.96	1.20
DiKTa	0.38	1.32

In naphthalene, the exchange coupling between the spin centers is the strongest, as both the HOMO and LUMO are similarly delocalized over the entire π-system. The spin-α and spin-β electrons in the diabatic states of naphthalene exhibit nearly identical spatial distributions, leading to almost vanishing spin densities (Figure 6, left) and minimal absolute spin populations. In contrast, the introduction of electron-donating and -accepting groups in PNA promotes spatial separation between the spin-α and spin-β electrons in the spin-permutation diabats (Figure 6, center), thereby significantly weakening the exchange coupling compared to naphthalene. Finally, the multiresonant atomic framework of DiKTa further enhances the localization of spin-α and spin-β electrons on different atomic sites (Figure 6, right), giving rise to the largest absolute spin populations and the weakest exchange coupling among the three chromophores.

**Figure 6:**
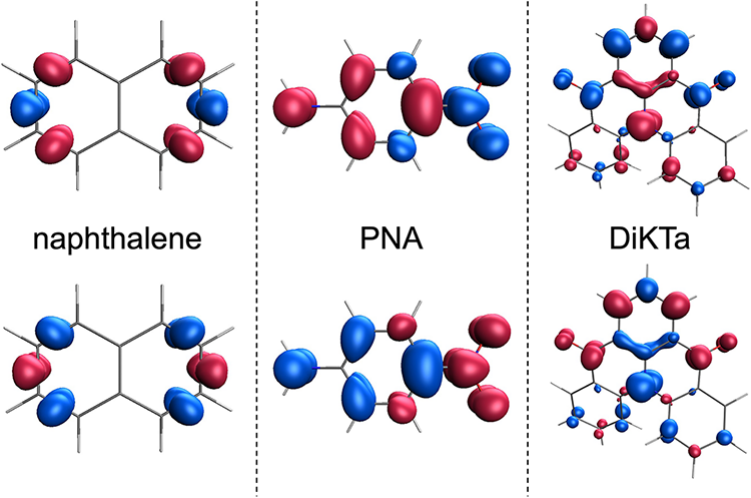
Spin density of the two diabatic states obtained from the diabatization of *T*_1_ and *S*_1_ of naphthalene (left), PNA (center), and DiKTa (right) molecules computed at the RAS­(*h*,*p*)-SF/6-31G level. Isovalue of 0.003 bohr^–3^.

### 4.7 Coupling of Spin-Triplet States

In this final example, we examine the coupling between two *S* = 1 states, specifically focusing on the interaction between two triplet excitations leading to the formation of spin-adapted triplet-pair (TT) states. The TT state is a multiexcitonic configuration arising from the coupling of two localized triplets, known to play a central role in SF^41^ and TTA^45^ photophysical processes. Here, we apply our diabatization scheme to a noncovalent tetracene dimer, a prototypical system well-known to undergo SF.^46–48^ Specifically, we employ a coplanar tetracene dimer geometry optimized at the scaled-opposite-spin (SOS) second-order Møller–Plesset perturbation theory (MP2) level.^49^ The diabatization is performed between the singlet and triplet TT states computed at the RAS-2SF/6-31G level, excluding hole and particle configurations and using the ROHF quintet as the reference configuration.

Figure 7 displays the spin densities of the two resulting diabatic states, showing a clear localization of the spin-α and spin-β densities on different monomers. Interestingly, the local triplets are not uniformly distributed across each tetracene unit but instead exhibit enhanced spin density on one side of the molecules. This asymmetry can be rationalized as a consequence of the intermolecular π–π interactions, which favor partial delocalization of the frontier orbitals across the dimer interface.

**Figure 7:**
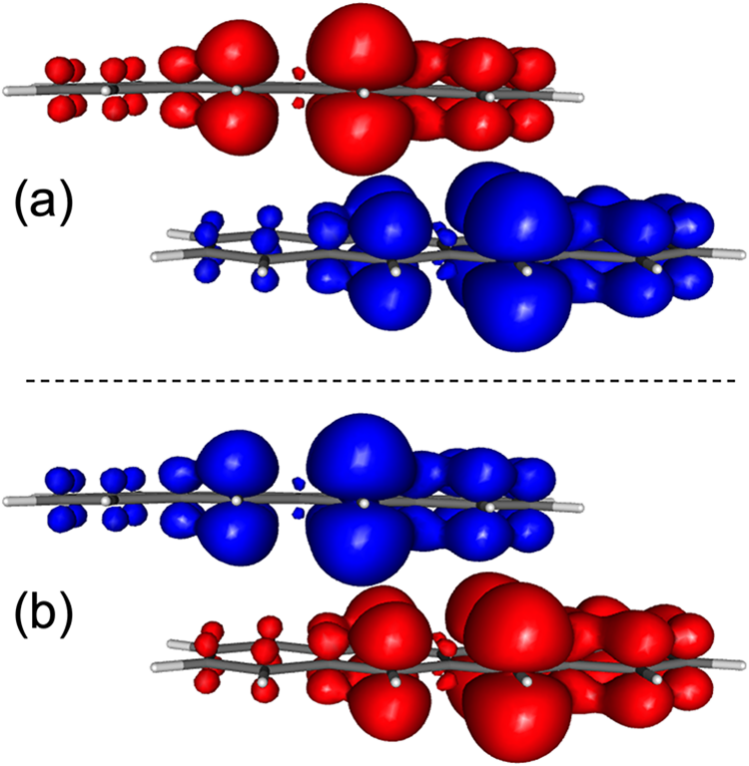
Spin density of the two diabatic states (a and b) obtained from the diabatization of singlet and triplet triplet-pair states computed for the coplanar tetracene dimer. Isovalue of 0.001 bohr^–3^.

We find that the absolute spin population of the two diabatic states is 3.24, noticeably smaller than the ideal value of 4 expected for two noninteracting triplet states. This deviation can be understood by considering that the diabatic states arise as linear combinations of the singlet and triplet triplet-pair (^1,3^TT) configurations. For pure (unmixed) ^1^TT and ^3^TT states, the corresponding spin-adapted wave functions can be expressed as

26 $${|}^{1}\mathrm{TT}\rangle =\frac{1}{\sqrt{3}}\left(\left|T_{+}T_{-}\right\rangle +\left|T_{-}T_{+}\right\rangle -\left|T_{0}T_{0}\right\rangle \right)$$

27 $${|}^{3}\mathrm{TT}\rangle =\frac{1}{\sqrt{2}}\left(\left|T_{+}T_{-}\right\rangle -\left|T_{-}T_{+}\right\rangle \right)$$

where T_+_, T_0_, and T_–_ denote the three *M*_*s*_ components of a triplet. When the diabatic states are formed as the symmetric and antisymmetric linear combinations of these singlet and triplet TT configurations

28 $$\left|\Xi_{\pm }\right\rangle =\frac{1}{\sqrt{2}}\left({|}^{1}\mathrm{TT}\rangle \pm {|}^{3}\mathrm{TT}\rangle \right)$$

the expected absolute spin population, *S*_abs_, can be estimated by accounting for the relative weights of the T_+_T_–_ and T_–_T_+_ components, each contributing two unpaired spins. From this consideration, one obtains *S*_abs_ = 3.27, in excellent agreement with the computed value (3.24) for the diabatic states of the tetracene dimer.

## 5 Conclusions

We have presented a general diabatization scheme based on the spin-permutation operator, designed to characterize molecular systems in terms of interacting electronic spins. This spin-permutation diabatization provides a direct mapping between ab initio electronic states and spin-effective Hamiltonians, avoiding the need for projection techniques or localized orbital definitions. The method allows (i) the decomposition of spin-pure eigenstates into spin-localized diabatic components and (ii) the evaluation of exchange coupling parameters in a physically transparent real-space representation.

The performance of the approach was demonstrated across a range of representative systems. For the ethylene torsion, the method captures the continuous evolution of spin interactions along the twisting coordinate, revealing the smooth transformation of diabatic spin densities and exchange couplings as the π-bond is broken. For the diradicals (benzynes, xylylenes, and methylene) the spin-diabatization accurately reproduces the spatial localization of unpaired electrons and rationalizes the relative singlet–triplet energy gaps in terms of the underlying spin–spin couplings. Application to the trimethylenebenzene triradical illustrates the ability of the formalism to describe systems with three interacting spins and to resolve competing coupling pathways leading to complex spin-state manifolds. In organic chromophores, the method allows a diabatic analysis of the lowest singlet and triplet excited states, providing a clear picture of their energy separation and spin composition. Finally, the tetracene dimer example demonstrates the applicability of the approach to multiexcitonic systems, where the spin-permutation diabatization identifies the diabatic triplet-pair configurations and quantifies their effective exchange interaction.

Overall, the proposed diabatization framework offers a conceptually transparent and computationally robust route to extract spin Hamiltonians directly from ab initio wave functions. Its general applicability to any number of interacting spins and compatibility with diverse electronic structure methods make it a valuable tool for studying magnetic interactions, excited-state spin dynamics, and spin-dependent photophysical processes in molecular systems. We expect this methodology to be particularly useful in cases where the distribution of unpaired electrons is far from trivial, such as in strongly correlated, multicenter, or delocalized spin systems.

## Supplementary Material



## Data Availability

The data that supports the findings of this study are available within the article and its Supporting Information, which contains additional and complementary data for the studied systems.

## Notes

Analysis of the diabatic states along the dissociation of H_2_, characterization of the dinuclear [Cu_2_Cl_6_]^2–^ complex, and molecular coordinates (PDF)
