# Supplementary material for: Spin-Permutation Diabatization: A General Framework for Spin Localization and Exchange Coupling
Source: J Chem Theory Comput. 2026 Jan 5;22(2):963–71. doi: 10.1021/acs.jctc.5c01904 (PMC12854749; doi:10.1021/acs.jctc.5c01904)
Supplement: Supplementary file 1 [file ct5c01904_si_001.pdf]

Supporting Information: Spin-Permutation Diabatization: A General Framework for Spin Localization and Exchange Coupling

Alicia Omist[†,‡] and David Casanova[*,†,¶]

†*Donostia International Physics Center (DIPC), 20018 Donostia, Euskadi, Spain*

‡*Polimero eta Material Aurreratuak: Fisika, Kimika eta Teknologia Saila, Kimika Fakultatea, Euskal Herriko Unibertsitatea (EHU), PK 1072, 20080 Donostia, Euskadi, Spain*

¶*IKERBASQUE, Basque Foundation for Science, 48009 Bilbao, Euskadi, Spain*

E-mail: david.casanova@dipc.org

Contents

S1 Convergence of the diabatization procedure **S2**

S2 Additional results **S3**

S2.1 H_2 dissociation S3

S2.2 The $[Cu_2Cl_6]^{2-}$ complex S5

S3 Molecular geometries **S6**

References **S13**

S1 Convergence of the diabatization procedure

The iterative nature of our spin diabatization procedure is closely analogous to established localization algorithms such as Boys-type diabatization or Boys orbital localization, which are routinely used to construct localized diabatic states for multiple electronic states. In these methods, one optimizes a well-defined localization functional by successive pairwise rotations of a unitary transformation matrix, in a manner directly analogous to orbital localization procedures.[S1]

Because the optimization targets a smooth objective function and operates over a compact rotation space, the convergence behavior in practice tends to be robust: the number of macro-iterations required to achieve localization scales roughly quadratically with the number of states, as each iteration applies all pairwise rotations between the active states. Convergence has been observed in a wide range of applications even for moderately large sets of states, and it is rare for localized diabatization procedures to fail to converge unless the underlying adiabatic states are nearly degenerate or not meaningfully distinct (i.e., they lack a definable diabatic character under the chosen metric).

There is no known intrinsic threshold in the number of states beyond which the algorithm systematically fails, nor is there an inherent limitation related to the magnitude of local magnetic moments (such as comparing spins of 1/2 versus higher spins). Any such difficulties would be analogous to those encountered in orbital localization (e.g., mediating near-linear dependencies in the localization functional) and can generally be ameliorated by standard techniques (better initial guesses, convergence controls on the localization metric, etc.).[S2] More generally, based on the long-established behavior of Boys diabatization and related orbital localization schemes, we do not expect intrinsic barriers to convergence arising solely from the number of states or from the magnitude of the local spins (e.g., spins larger than 1/2). The algorithm optimizes a well-behaved localization functional, and non-convergence is rare unless the underlying electronic states are nearly linearly dependent or physically indistinguishable, conditions under which any diabatization becomes ill-defined.

S2 Additional results

S2.1 H_2 dissociation

As a minimal two-electron system, the H_2 molecule provides a simple framework for illustrating the spin-permutation diabatization scheme. We apply the diabatization method between the singlet ground state and the low-lying triplet state to analyze the σ-bond dissociation as a function of different internuclear distance.

At the equilibrium distance, 0.74 Å, the S_0 state is described by the doubly occupancy of the σ-bonding orbital arising from the overlap of the 1s orbitals of each hydrogen. This overlap leads to a large energy separation between the corresponding singlet and triplet adiabatic states, reflecting the stabilization of the bonding configuration and a strong spin-exchange coupling. As the interatomic distance increases, the overlap between the atomic orbitals diminishes, leading to a gradual reduction in the singlet–triplet energy gap and so a decrease in the magnitude of magnetic interaction. At large separations, e.g., 5.0 Å, the system behaves as a two non-interacting $S = \frac{1}{2}$ spin-centers and the spin-exchange coupling value approaches to zero.

The computed diabatic states for each internuclear distance correspond to the localized spin functions $|\alpha\beta\rangle$ and $|\beta\alpha\rangle$, each assigning opposite spins to the two hydrogen atoms.

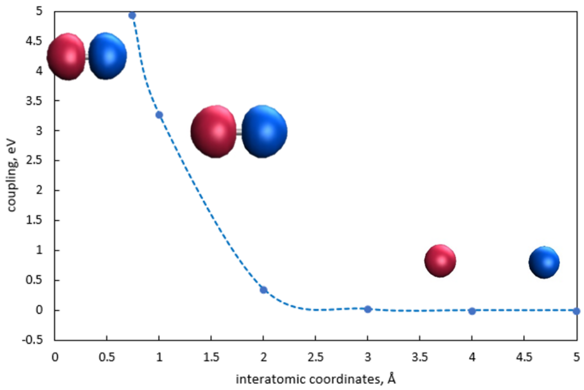

Figure S1: Electronic coupling (in eV) between the two diabatic states from the S_0 and T_1 along the hydrogen interatomic coordinate. Inset: spin density of one of the diabatic states at 0.74 Å, 1.0 Å, and 5.0 Å. The other diabat present the equivalent spin density distribution with interchanged $\alpha - \beta$ densities. Isovalue: 0.02 bohr^{-3}.

S2.2 The $[Cu_2Cl_6]^{2-}$ complex

As a proof-of-concept of the application of the spin-permutation diabatization method, here we characterize the diabatic state obtained from the ground state triplet and first excited singlet state in a copper dinuclear complex: $[Cu_2Cl_6]^{2-}$.

For that, we employ RAS(h, p)-SF with the 6-31G(d) basis set, Here we consider a minimal RAS2 space with 2 electrons in the 2 frontier orbitals of the triplet reference configuration obtained at the restricted open-shell Kohn-Sham (ROKS) level with the B3LYP exchange-correlation functional and the 6-31G(d). The choice of has been motivated for the to converge to the lowest triplet state con and to introduce electron correlation effects and the orbital level in order to provide a better description of the Cu−Cl bond. The obtained spin densities of the two diabatic states are shown in Figure S2.

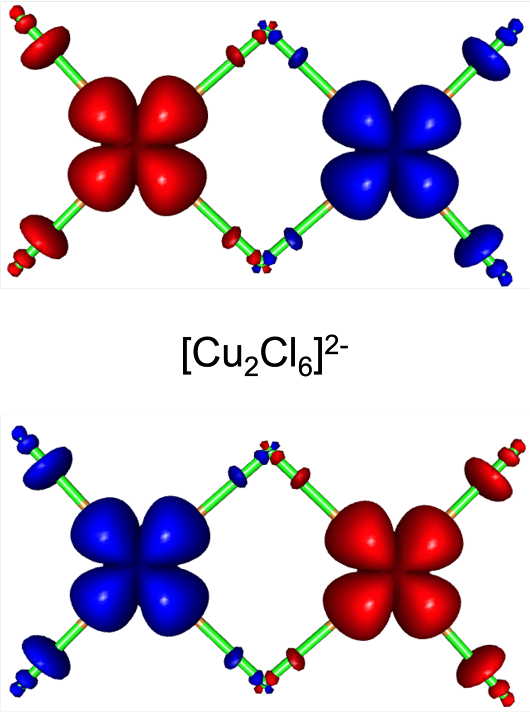

Figure S2: Diabatic states of $[Cu_2Cl_6]^{2-}$ computed at the RAS(h, p)-SF/6-31G(d) level. Molecular structure obtained from reference S3.

S3 Molecular geometries

Table S1: Molecular cartesian coordinates (in Å) of ethylene optimized at the RHF/6-31G* computational level.

C	0.65846050	-0.00000001	0.00000000
C	-0.65846050	-0.00000006	0.00000000
H	1.22566614	0.91423547	0.00000000
H	1.22566616	-0.91423548	0.00000000
H	-1.22566615	0.91423546	0.00000000
H	-1.22566615	-0.91423549	0.00000000

Table S2: Molecular cartesian coordinates (in Å) of the broken-symmetry state of *o*-benzyne optimized at the UB3LYP/6-31G* computational level.

C	0.000000	0.626238	-1.228690
C	0.000000	-0.626238	-1.228690
C	0.000000	1.448484	-0.113362
C	0.000000	-1.448484	-0.113362
C	0.000000	0.703267	1.066171
C	0.000000	-0.703267	1.066171
H	0.000000	2.519674	-0.117121
H	0.000000	-2.519674	-0.117121
H	0.000000	1.218692	2.008740
H	0.000000	-1.218692	2.008740

Table S3: Molecular cartesian coordinates (in Å) of the broken-symmetry state of *m*-benzyne optimized at the SF-TDDFT/B5050LYP/cc-pVTZ computational level.[S4]

C	-0.6804935700	-0.9934314339	-0.0000136727
C	-1.5919504832	0.0003016204	-0.0000011662
H	-2.6617084775	0.0004866197	-0.0000356336
C	-0.6801509685	0.9937493002	-0.0000024980
C	0.6682311816	1.1607973471	-0.0000062013
H	1.1076641699	2.1400289960	-0.0000548499
C	1.4254196489	-0.0002104031	0.0000283463
H	2.5025092016	-0.0003939165	-0.0000120058
C	0.6678271763	-1.1609635391	-0.0000019555
H	1.1069180702	-2.1403465405	-0.0000594211

Table S4: Molecular cartesian coordinates (in Å) of the broken-symmetry state of *p*-benzyne optimized at the UB3LYP/6-31G* computational level.

C	0.000000	0.000000	1.321929
C	0.000000	-0.000000	-1.321929
C	0.000000	1.227416	0.698952
C	0.000000	-1.227416	0.698952
C	0.000000	1.227416	-0.698952
C	0.000000	-1.227416	-0.698952
H	0.000000	2.144550	1.256066
H	0.000000	-2.144550	1.256066
H	0.000000	2.144550	-1.256066
H	0.000000	-2.144550	-1.256066

Table S5: Molecular cartesian coordinates (in Å) of the broken-symmetry state of *o*-xylylene optimized at the UHF/6-31G* computational level.

C	-0.60107158	-0.73241013	-0.00001228
C	-1.76791923	-1.49732649	0.00004248
H	-2.74983297	-1.06774959	0.00019867
H	-1.70951398	-2.57023899	0.00013009
C	0.66543531	-1.39989084	-0.00005183
H	0.67159701	-2.47560819	-0.00008908
C	1.86836162	-0.70645496	-0.00003112
H	2.79891379	-1.24502412	-0.00006229
C	1.86836161	0.70645504	0.00003090
H	2.79891374	1.24502427	0.00006086
C	0.66543523	1.39989081	0.00005137
H	0.67159679	2.47560816	0.00009864
C	-0.60107163	0.73241006	0.00001159
C	-1.76791924	1.49732649	-0.00004312
H	-2.74983299	1.06774960	-0.00019811
H	-1.70951395	2.57023898	-0.00012669

Table S6: Molecular cartesian coordinates (in Å) of the triplet ground state of m-xylylene optimized at the UHF/6-31G* computational level.

C	1.24491622	0.33652911	-0.00012497
C	2.46229035	1.03639651	-0.00007008
H	2.48730894	2.11060693	0.00070493
H	3.40297880	0.51708877	0.00079649
C	-0.00000019	1.02490821	-0.00016180
H	-0.00000026	2.10111821	-0.00019605
C	-1.24491645	0.33652885	-0.00016422
C	-2.46229084	1.03639579	-0.00011304
H	-2.48730974	2.11060598	0.00091827
H	-3.40297885	0.51708770	0.00098678
C	-1.21493123	-1.09504315	-0.00001377
H	-2.14301118	-1.63851675	0.00004789
C	0.00000066	-1.78398955	0.00005607
H	0.00000085	-2.85956955	0.00014956
C	1.21493145	-1.09504324	0.00000970
H	2.14301159	-1.63851650	0.00008483

Table S7: Molecular cartesian coordinates (in Å) of the broken-symmetry state of p-xylylene optimized at the UHF/6-31G* computational level.

C	0.00000448	-0.00000001	1.43977168
C	0.00000422	-0.00000001	2.83256768
H	-0.00006551	0.91831443	3.39068015
H	-0.00005433	-0.91831447	3.39068013
C	0.00000415	-1.22460839	0.68863548
H	0.00000368	-2.15826238	1.22332812
C	0.00000415	-1.22460839	-0.68863548
H	0.00000368	-2.15826238	-1.22332812
C	0.00000448	-0.00000001	-1.43977168
C	0.00000422	-0.00000001	-2.83256768
H	-0.00005433	-0.91831447	-3.39068013
H	-0.00006551	0.91831443	-3.39068015
C	0.00000537	1.22460841	-0.68863547
H	0.00000584	2.15826235	-1.22332820
C	0.00000537	1.22460841	0.68863547
H	0.00000584	2.15826235	1.22332820

Table S8: Molecular cartesian coordinates (in Å) of the triplet ground state of methylene optimized at the UHF/6-31G* computational level.

C	0.0000000000	0.0000000000	0.0071654834
H	-0.9946482237	0.0000000000	-0.4158032910
H	0.9946482237	0.0000000000	-0.4158032910

Table S9: Molecular cartesian coordinates (in Å) of the quartet ground state of TMB optimized at the UHF/6-31G computational level.

C	-0.71905042	-1.24749070	0.00000000
C	-1.42225541	-2.46748972	0.00000000
H	-2.49521290	-2.49145072	0.00000000
H	-0.90518030	-3.40793920	0.00000000
C	0.70721996	-1.22292023	0.00000000
H	1.24522846	-2.15324107	0.00000000
C	1.43988487	0.00102953	0.00000000
C	2.84803751	0.00203639	0.00000000
H	3.40526636	-0.91519200	0.00000000
H	3.40395410	0.92006071	0.00000000
C	0.70547040	1.22393029	0.00000000
H	1.24214793	2.15501956	0.00000000
C	-0.72083365	1.24646114	0.00000000
C	-1.42578250	2.46545335	0.00000000
H	-2.49877315	2.48787999	0.00000000
H	-0.91005270	3.40664126	0.00000000
C	-1.41269094	-0.00101006	0.00000000
H	-2.48737667	-0.00177850	0.00000000

Table S10: Molecular cartesian coordinates (in Å) of naphthalene optimized at the RHF/6-31G* computational level.

C	-0.00000000	-0.70456490	0.00000000
C	0.00000000	0.70456490	0.00000000
C	-1.24284913	-1.39257606	0.00000000
H	-1.24036465	-2.46864819	0.00000000
C	-2.41618024	-0.70829512	0.00000000
H	-3.35217730	-1.23773084	0.00000000
C	-2.41618024	0.70829512	0.00000000
H	-3.35217730	1.23773084	0.00000000
C	-1.24284913	1.39257606	0.00000000
H	-1.24036465	2.46864819	0.00000000
C	1.24284913	-1.39257606	0.00000000
H	1.24036465	-2.46864819	0.00000000
C	2.41618024	-0.70829512	0.00000000
H	3.35217730	-1.23773084	0.00000000
C	2.41618024	0.70829512	0.00000000
H	3.35217730	1.23773084	0.00000000
C	1.24284913	1.39257606	0.00000000
H	1.24036465	2.46864819	0.00000000

Table S11: Molecular cartesian coordinates (in Å) of *p*-nitroaniline (PNA) optimized at the B3LYP/6-31G* computational level.

C	0.00000000	0.00000000	2.07462441
C	1.21587735	0.00000000	1.35413138
C	1.21552257	0.00000000	-0.03008269
C	0.00000000	0.00000000	-0.72009673
C	-1.21552257	0.00000000	-0.03008269
C	-1.21587735	0.00000000	1.35413138
H	2.15860772	0.00000000	1.89528018
H	2.14227205	0.00000000	-0.59095517
H	-2.14227205	0.00000000	-0.59095517
H	-2.15860772	0.00000000	1.89528018
N	0.00000000	0.00000000	3.44238912
H	0.86124475	0.00000000	3.96441037
H	-0.86124475	0.00000000	3.96441037
N	0.00000000	0.00000000	-2.17397268
O	1.09088543	0.00000000	-2.75294114
O	-1.09088543	0.00000000	-2.75294114

Table S12: Molecular cartesian coordinates (in Å) of DiKTa optimized at the B3LYP-D3BJ/Def2-SVP computational level.

C	0.00006943	3.90419526	0.00009514
C	-1.17550386	3.19809623	0.24753781
C	-1.17550386	3.19809623	0.24753781
C	-1.19567157	1.79771056	0.23731002
C	0.00001626	1.07923171	0.00000969
C	1.19573196	1.79767963	-0.23724351
C	1.17561651	3.19806678	-0.24738750
C	-2.47186553	1.09044570	0.47335920
C	-2.44242172	-0.34568569	0.11901188
C	-1.22675118	-0.99837967	-0.18477231
N	-0.00001088	-0.31813191	-0.00003641
C	1.22670210	-0.99844038	0.18465577
C	2.44239967	-0.34577205	-0.11907569
C	2.47190056	1.09038128	-0.47332864
C	-3.69877717	-2.33153861	-0.49799005
C	-2.49780404	-2.94920253	-0.87836226
C	-1.27936020	-2.29610079	-0.73145730
C	1.27925738	-2.29620404	0.73124549
C	2.49767484	-2.94936359	0.87811065
C	3.69867432	-2.33171811	0.49779165
C	3.66177957	-1.02982718	0.02154567
H	-2.12010691	3.70468934	0.45546685
H	2.12023896	3.70463675	-0.45528484
H	4.56907853	-0.48052359	-0.23652085
H	2.51104327	-3.95222351	1.31160708
H	0.36638416	-2.78581075	1.06386103
H	-0.36650818	-2.78571830	-1.06411545
H	-2.51121384	-3.95203005	-1.31193224
H	-4.56910505	-0.48036302	0.23646047
O	3.48719172	1.66114946	-0.84489910
O	-3.48713236	1.66122882	0.84497286
C	-3.66182899	-1.02968342	-0.02165058
H	-4.64943009	-2.85710188	-0.60817393
H	4.64930634	-2.85732575	0.60794444
H	0.00009000	4.99592832	0.00012698

Table S13: Molecular cartesian coordinates (in Å) of a non-covalent coplanar tetracene dimer optimized at the SOS-MP2/def2-SV(P) computational level.[S5]

C	-3.0163731882	-0.0662263444	1.4588357065
C	-4.2548412373	-0.2210608843	0.8265267294
C	-4.3763752987	-1.1359635732	-0.2898214152
C	-3.2558412352	-1.8630721629	-0.7078797525
C	-1.9948079996	-1.7056476889	-0.0798514767
C	-1.8722500658	-0.7839204215	1.0308070731
C	-0.6048401749	-0.6087761741	1.6443054463
C	0.5198455876	-1.3195722862	1.2140377494
C	1.8149453490	-1.1418682204	1.8274105184
C	2.9043299989	-1.8546393833	1.3918767089
C	2.7746792670	-2.7997234766	0.3106587951
C	1.5629754323	-2.9934782635	-0.3048723211
C	0.3935548486	-2.2560840330	0.1139028629
C	-0.8500051334	-2.4306247932	-0.5014619687
H	-2.9226280486	0.6337093490	2.3009267948
C	-5.4175654804	0.5301624061	1.2360366347
C	-5.6577557338	-1.2655019390	-0.9428863178
H	-3.3493218662	-2.5643821259	-1.5500262695
H	-0.5101987338	0.0981490625	2.4806988862
H	1.9116966142	-0.4232678443	2.6522049716
H	1.4637339091	-3.7130836411	-1.1295537307
H	-0.9459755962	-3.1416290957	-1.3352443427
C	-6.7393235865	-0.5281071217	-0.5266046208
C	-6.6168971566	0.3851659198	0.5820474519
H	-5.3239247101	1.2231231462	2.0834423281
H	-7.4931955145	0.9638224388	0.9026389579
H	-5.7507960709	-1.9641474074	-1.7858071603
H	-7.7070801512	-0.6338188393	-1.0342246110
C	0.8848475191	2.4461599374	0.5219237906
C	-0.3725950336	2.2866589897	-0.0689817868
C	-0.5303712400	1.3581784732	-1.1719553621
C	0.5771014780	0.6349610729	-1.6248893660
C	1.8569369426	0.7900988956	-1.0323229589
C	2.0131904889	1.7099221855	0.0757006914

C	3.2884578577	1.8507555874	0.6784789765
C	4.3896248643	1.1052555748	0.2415867330
C	5.6846706852	1.2150678327	0.8709354536
C	6.7459287290	0.4579144627	0.4379930086
C	6.5884170517	-0.4566182472	-0.6651814038
C	5.3753400987	-0.5830744217	-1.2971587538
C	4.2331875495	0.1891761509	-0.8694035138
C	2.9815093919	0.0536786813	-1.4794419474
H	1.0047429618	3.1528752220	1.3562774376
C	-1.5273701726	3.0296373457	0.3798989156
C	-1.8374872903	1.2014763772	-1.7653238071
H	0.4584654469	-0.0672456821	-2.4621667364
H	3.4082173948	2.5509126876	1.5182539257
H	5.8047296050	1.9144802071	1.7097832349
H	5.2546259674	-1.2770378935	-2.1403233945
H	2.8613763593	-0.6455576146	-2.3188126176
C	-2.9113474852	1.9206506294	-1.3029551372
C	-2.7522635201	2.8530117178	-0.2144498311
H	-1.4057335333	3.7408265555	1.2088928882
H	-3.6243457199	3.4200558956	0.1374855619
H	-1.9566764207	0.4919849682	-2.5949691926
H	-3.9024190125	1.7852535559	-1.7550278133
H	3.8856118887	-1.7042736104	1.8603130826
H	3.6585955531	-3.3619599167	-0.0184742777
H	7.4488257163	-1.0512695859	-0.9992952593
H	7.7242813138	0.5486433392	0.9278783067

References

(S1) Subotnik, J. E.; Yeganeh, S.; Cave, R. J.; Ratner, M. A. Constructing diabatic states from adiabatic states: Extending generalized Mulliken–Hush to multiple charge centers with Boys localization. *J. Chem. Phys.* **2008**, *129*, 244101.

(S2) Jansík, B.; Høst, S.; Kristensen, K.; Jørgensen, P. Local orbitals by minimizing powers of the

orbital variance. *J. Chem. Phys.* **2011**, *134*, 194104.

(S3) Castell, O.; Miralles, J.; Caballol, R. Structural dependence of the singlet-triplet gap in doubly bridged copper dimers: a variational CI calculation. *Chemical Physics* **1994**, *179*, 377–384.

(S4) Orms, N.; Rehn, D. R.; Dreuw, A.; Krylov, A. I. Characterizing Bonding Patterns in Diradicals and Triradicals by Density-Based Wave Function Analysis: A Uniform Approach. *Journal of Chemical Theory and Computation* **2018**, *14*, 638–648, PMID: 29268010.

(S5) C. A. Valente, D.; do Casal, M. T.; Barbatti, M.; Niehaus, T. A.; Aquino, A. J. A.; Lischka, H.; Cardozo, T. M. Excitonic and charge transfer interactions in tetracene stacked and T-shaped dimers. *J. Chem. Phys.* **2021**, *154*, 044306.
